# Waste Rubber from End-of-Life Tires in ‘Lean’ Asphalt Mixtures—A Laboratory and Field Investigation in the Arid Climate Region

**DOI:** 10.3390/polym13213802

**Published:** 2021-11-03

**Authors:** Stefano Marini, Michele Lanotte

**Affiliations:** Department of Civil Infrastructure and Environmental Engineering, Khalifa University of Science and Technology, Abu Dhabi 127788, United Arab Emirates; stefano.marini@ku.ac.ae

**Keywords:** rubberized asphalt, lean mixtures, permanent deformation, cracking performance, IRI, friction, sound absorption

## Abstract

Waste rubber from end-of-life tires has been proved to be an excellent source of polymeric material for paving applications. Over the years, however, the rubberized asphalt technology has never been used in ‘lean’ (low bitumen content) asphalt mixtures typically used in arid regions. This study offers an insight on the potential benefits and drawbacks resulting from this technology if applied in such ‘lean’ mixes. Results show that the ‘lean’ nature of those asphalt mixes eliminates the potential benefits given by the modified bitumen for rutting performance. Instead, the aggregates gradation plays a major role in the response of the materials, with gap-graded mixtures often outperforming those with a dense-graded gradation. On the contrary, fatigue cracking resistance is affected by the bitumen properties, and rubberized asphalt perform better than others. The performance-based analysis suggests that the current specifications tend to overachieve the goal of reducing permanent deformation while cracking becomes a major concern which can be solved by using rubberized asphalt. In the field, gap-graded asphalt with rubberized bitumen showed the best response in terms of skid resistance and noise reduction.

## 1. Introduction

Crumb rubber (CR) from end-of-life tires has been used as an additive in asphalt pavements since the 1950s. Two techniques have been worldwide recognized to include CR in asphalt mixtures, namely ‘wet process’ and ‘dry process’ [1]. Bitumen modified with the ‘wet process’ has been reported to enhance rutting and cracking resistance of asphalt mixtures significantly [2,3,4,5,6,7]. Moreover, several studies demonstrated the benefits of such technology from an environmental perspective with some health issues that might arise during construction for the paving crew [8,9,10,11]. During the production process of CR-modified bitumen (CRMB), the combined action of high blending temperature (170–190 °C) and mechanical agitation promotes the diffusion of the oily fraction of the bitumen into the cross-linked polymeric network of the rubber. As a result, each rubber particle swells up to 2–3 times its original size due to the formation of a layer of gel-like material around the core of original rubber [12]. The presence of such swollen particles is both an advantage and a significant drawback of the ‘wet technology’. The exceptional elastic response of the swollen rubber particles in the bitumen makes the mixtures highly resistant to the accumulation of permanent deformations. Also, the reduced oily fraction in the base bitumen available to react in oxidation and volatilization phenomena produces mixes that are less prone to aging. On the other side, the swelling of rubber produces a simultaneous reduction of the inter-particle distance and the hardening of the base bitumen, which leads to an overall increase of viscosity [13]. The latter is a significant concern during pumping, transportation, mixing, and lay-down phases.

Given the physical peculiarities of the rubberized bitumen, mixtures were initially designed according to reference formulations, in which a non-continuous aggregate size distribution, Gap-Graded (GG) or Open-Graded (OG) was combined with binder contents, higher than those of the standard Dense-Graded (DG) mixtures [14,15,16,17,18,19]. Residual air voids in these GG- and OG-mixes tend to be in the form of large air inclusions rather than small and finely distributed within the bulk material, as in the case of DG mixes. Such discontinuities create the necessary room for the modified binder and promote the formation of a multitude of stone-to-stone contact points, which contribute to a better distribution of stresses under loading [14]. The missing fraction of aggregates also leads to a better macrotexture after compaction and provides excellent skid resistance [20].

Even though the benefits of using these gradations are well documented, DG rubberized mixtures were also studied, and eventually implemented, in those locations where environmental conditions and construction practices did not allow the use of GG and OG Hot-Asphalt Mixtures (HMAs). Given the high-viscosity of the rubberized binder and the presence of rubber particles in the bituminous matrix, the rubberized DG HMAs have a slightly higher bitumen content compared to DG mixtures with either neat or other polymer-modified binders. In most of the cases, it has been found that the superior performance of the rubberized bitumen improved the thermo-mechanical response of the mixtures compared to traditional HMAs [21,22].

The excellent experimental and practical results documented in the scientific literature make the use of rubber for paving applications an interesting solution for many countries, and specifically for those that are experiencing substantial expansion of their transportation infrastructure network. However, the implementation of new technologies within the framework of local specifications is not always straightforward. This statement is particularly relevant in the Middle East, where severe climate conditions pose a great challenge to pavement materials and structures. The United Arab Emirates is no exception in this scenario. Local governmental agencies, in fact, have modified the traditional Marshall volumetric requirements to obtain mixtures that better perform in terms of rutting and bleeding, two of the major distresses in the area due to high service temperatures and heavy loads [23]. As a result, the binder content of the HMAs produced locally never exceeds 4%, with typical values ranging from 3.6 to 4% for base, intermediate, and wearing courses. Such values are common in the United States and other countries for ATBs (Asphalt Treated Bases) rather than HMAs. The results presented in this paper are part of a research project aimed at investigating the potential benefits of using asphalt rubber in dense- and gap-graded asphalt mixtures within the framework of the current Abu Dhabi technical standards. A full-scale test section was constructed on an existing major truck-road (Highway E30) designed to withstand more than 100 million ESALs over its service life. Five mixtures were designed and then sampled in the field during construction. Cracking, rutting, moisture damage and sound absorption were investigated in the laboratory while roughness, surface texture, and skid resistance were evaluated in the field. Those measurements were performed during the winter season in Abu Dhabi (approximate mean air temperature of 25 °C) after 14 months from construction during which the pavement structure has been subjected approximately to 6 million ESALs with the intend of providing information about the status of the pavement sections at an early stage of service life.

## 2. Field Test Section

**Figure 1:**
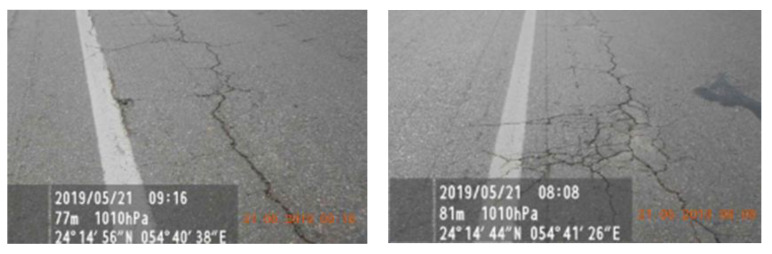
Field survey of the existing pavement structure.

The existing pavement structure presented severe cracking damage on the truck lane. Cracks were mostly longitudinal along the right-wheel path. However, some areas with interconnected cracks in an advanced state of propagation were also found (Figure 1).

The top 12 cm of bituminous materials were milled and replaced with:

1. 7 cm of HMA as intermediate course (PG64-22) and,
2. 5 cm of the following HMAs as wearing course:
  - Dense-graded HMA with neat bitumen PG64-10 (DG-NB),
  - Dense-graded HMA with SBS-modified bitumen PG82-22 (DG-PMB),
  - Dense-graded HMA with rubberized bitumen PG76-22 (DG-CRMB),
  - Gap-graded HMA with SBS-modified bitumen PG82-22 (GG-PMB), and
  - Gap-graded HMA with rubberized bitumen PG76-22 (GG-CRMB).

The final length of the test section was 2.2 km, with 200 m of DG-NB and the remaining length of the test section equally distributed among the other mixtures.

## 3. Preliminary Characterization of Materials and Mix-Design Results

### 3.1. Asphalt Binders and Aggregates

The PMB and CRMB used in this study were produced locally by using the same base bitumen PG58-10. The PMB is classified as PG82-22, Continuous High PG 84.4, while the CRMB is a PG 76-22, Continuous High PG 79.4. The neat bitumen used to produce the dense-graded asphalt mixture is a PG64-10, Continuous High PG 67.7, commonly used in the region.

**Figure 2:**
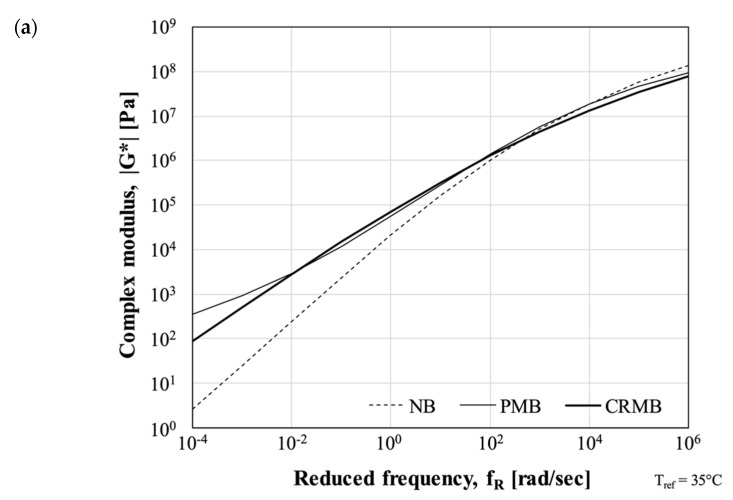

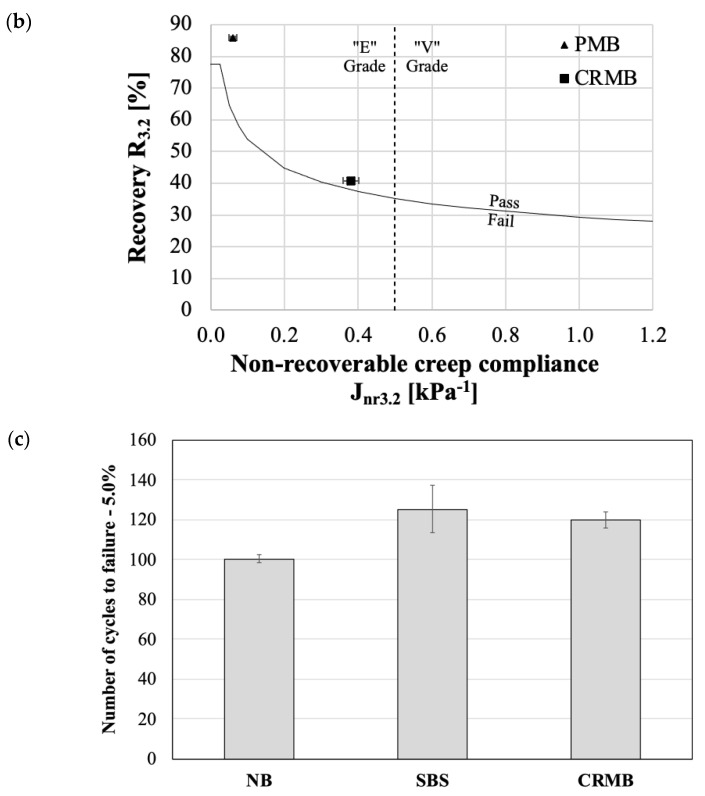
Results of Asphalt Binders: (**a**) Master Curves; (**b**) MSCR; (**c**) LAS.

All binders were initially subjected to linear viscoelastic analysis. Dynamic shear rheometer (DSR) frequency sweep tests were conducted using the 25- and 8-mm parallel plates measuring systems at six temperatures (5, 20, 35, 55, 65, and 80 °C) and frequencies within the range 0.1–100 rad/sec. Strain levels were defined to perform the analysis in the linear viscoelastic response region. Complex modulus values (|G*|) were mathematically treated to build the master curve through the application of the time-temperature superposition (TTS) principle (Figure 2). The following models were selected for fitting master curve and shift factors [24]:

(1) $$log\left(\left|G^{*}\right|\right)=\delta +\frac{\alpha }{{\left[1+\lambda exp\left(\beta +\gamma log\left(f_{R}\right)\right)\right]}^{1/\lambda }}$$

(2) $$log\left(a_{T}\left(T\right)\right)=a_{1}\left(T^{2}-T_{ref}^{2}\right)+a_{2}\left(T-T_{ref}\right)$$

where: *δ*, *α*, *β*, *γ*, and *λ* are the coefficients of the generalized logistic sigmoidal model, *f_R_* is the reduced frequency (*f_R_* = *f·a(T)*), *T* and *T_ref_* are temperature and reference temperature (35 °C in this study), *f* is the frequency, *a*(*T*) is the shift factor, and *a*_1_ and *a*_2_ the coefficients of the second order polynomial equation for shift factors-temperature relationship. 

Binders were also subjected to multiple stress-creep recovery (MSCR) tests at 64 °C and linear amplitude sweep (LAS) at 20 °C. Following the procedure of the AASHTO M332 specification, MSCR tests were carried out by imposing two stress levels (0.1 kPa and 3.2 kPa). For each of them, the percentage recovery (R_0.1_ and R_3.2_) and non-recoverable creep compliance (J_nr0.1_ and J_nr3.2_) were calculated [25]. A synthetic graphical assessment of the MSCR is provided by correlating the J_nr3.2_ and R_3.2_ values (Figure 2). The LAS tests were performed in accordance with the AASHTO TP101-14 specification [26]. The DSR 8-mm parallel plates geometry with a 2-mm gap setting was first used for a frequency sweep tests in order to determine the complex modulus and phase angle of the asphalt binder (fingerprint test). The same sample was then subjected to a series of oscillatory load cycles at linearly increasing amplitude levels (0–30%) at a constant frequency of 10 Hz. The continuum damage approach, as described in the AASHTO TP101-14, was used to calculate the fatigue resistance in terms of number of loading cycles to failure (N_f_) at the 35% damage.

As previously mentioned, the performance of CRMBs depend significantly on the degree of interaction between the rubber particles and the asphalt binder. In this project, in order to minimize potential issues related to the common high viscosity of CRMBs, rubber particles reacted with the bitumen up to the degradation phase. As such, the CRMB did not include fully swollen rubber particles and appeared as a homogeneous bitumen-polymer blend. As expected, the results of the rheological characterization reported in Figure 2 indicate that the CRMB thus produced, does not provide a response in terms of rutting as good as the PMB. It is worth noticing that, despite a grade of difference in the traditional PG classification between the SBS- and CR-modified binders, both of them can be classified as PG64E in the PG+ classification. On the other side, the advanced stage of interaction between the rubber particles and the bitumen do enhance the fatigue cracking performance of the CRMB bitumen which is comparable to the one of the PMB.

**Figure 3:**
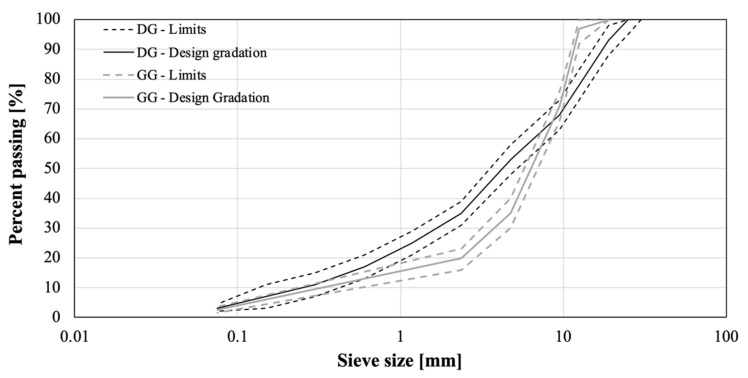
Aggregate gradation and limits.

The physical characteristics of the aggregates used in the mix-design process are reported in Table 1, while the design gradation of the DG and GG blends is shown in Figure 3.

### 3.2. Mix-Design Results

The five surface course mixes were designed with the Marshall methodology in agreement with the local specifications [27]. Volumetric and mechanical characteristics of the five mixtures are reported in Table 2. As expected, both DG- and GG-CRMB mixtures present a slightly higher optimum bitumen content compared to other mixes with the same gradation. The CRMB, in fact, includes rubber particles, which do not contribute to the lubricating action of the bitumen during compaction. Thus, for a given temperature, a slightly higher bitumen content is needed to achieve the desired level of compaction. Since no local specifications were available for GG-mixtures, the optimal job-mix formulae were found by fulfilling the same air voids, VMA, and VFA requirements of the DG mixtures.

## 4. Field and Laboratory Testing Methods

### 4.1. Laboratory Tests Methodologies

**Figure 4:**
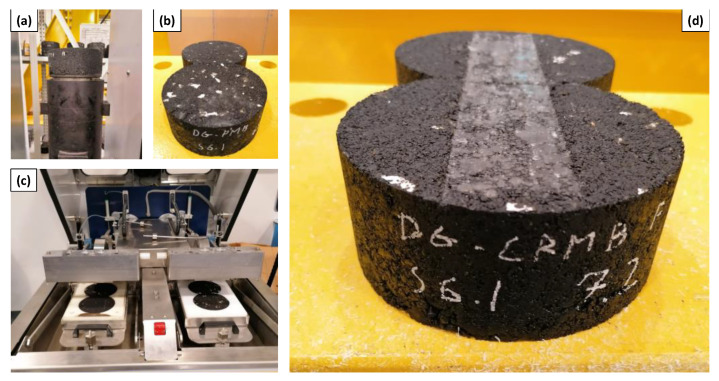
HWT sample preparation and testing: (**a**) GSC sample, (**b**) couple of samples with straight face by side, (**c**) HWT setup, (**d**) sample after test.

Resistance to permanent deformation and moisture damage of the five asphalt mixtures was evaluated by performing Hamburg Wheel Tracking (HWT) tests, in compliance with AASHTO T324 [28]. Cylindrical specimens of 150 mm diameter and 60 mm height were compacted in the Superpave Gyratory Compactor (SGC) with a target air void content of 7 ± 0.5%. Two SGC specimens were used for each HWT replicate. Each of them was preliminary cut in order to offer a straight contact surface to the other specimen and create a continuous path for the wheel tracking device. The test was conducted in wet conditions at a temperature of 60 ± 0.5 °C and the frequency of the moving wheel load was set to 30 passes per minute. At least two replicates per each mixture were performed (Figure 4).

The typical HMA behavior during the HWTT in presence of water can be divided into three phases: (i) post-compaction, (ii) creep, and (ii) stripping. The post-compaction phase consists of the densification of the mixture with a sudden decrease in air voids usually happening within the first 1000 cycles. The creep phase is the result of the viscous deformation of the asphalt mixture, and it is represented by an approximately constant rate of increase in rut depth. The last phase starts once the bonding between the asphalt binder and the aggregate degrades due to the presence of water.

Given the Abu Dhabi climate conditions, however, moisture damage is unlikely. Hence, the HWT device was upgraded with an air temperature controller and tests were also conducted in dry conditions at 60 ± 0.5 °C. A cylindrical sample of the same dimensions of the ones tested was instrumented with a thermocouple and inserted in the HWT device to control the material’s temperature during the test. 

Semi-Circular Bending (SCB) tests were used to characterize the cracking resistance of the mixtures, in compliance with the AASHTO TP124-18 [29]. SCB samples were obtained from Superpave Gyratory Compactor (SGC) specimens compacted at a height of 180 mm and a diameter of 150 mm. From the center of the SGC specimens, two cylindrical 50 mm thick slices are obtained and then cut into two identical halves. The target air voids of each SCB sample was set to 7 ± 1%. A notch is then cut using a tile saw along the axis of symmetry of each semi-circular specimen. Four replicates per each mixture were carried out in the Asphalt Mixture Performance Tester (AMPT) at 20 °C and a constant deformation rate of 50 mm/min. The output of the test is a load-deformation curve from which a number of fracture energy-related parameters can be obtained. Seitllari et al. showed that the hierarchy between mixtures is the same regardless of the parameter considered [30]. Moreover, the hierarchy between materials given by these parameters is the same as that provided by fatigue cracking tests in oscillatory mode at different strain levels [30]. In the same study, it was reported that the Flexibility Index (FI) [31], the Fracture Strain Tolerance (FST) [32], the Cracking Resistance Index (CRI) [33], and the N_flex_ factor [34] have acceptable repeatability. Thus, these parameters were considered in this study.

The normal incidence sound absorption coefficient was evaluated in the lab on cores collected from the test sections after construction. The Impedance Tube Method was used to test three cores per each mixture in the range of frequencies between 200 and 3150 Hz, in accordance with the procedure specified in the ISO 10534-2:1998E [35]. In this, a sound source generates plane waves, and their pressure is captured by two microphones placed at 45 mm far from each other. The distance from the sample to the closest microphone is 100 mm, while the total tube length is 470 mm. The maximum-length sequence (MLS) signal was used for this study.

### 4.2. Field Tests Methodologies and IFI calculation

The Mean Profile Depth (MPD) was measured by an automatic measurement system based on laser displacement sensors. The device was equipped with laser sensors, accelerator, and gyroscope to gather information about the actual sensors distance from the pavement surface and driving speed. The sensors can collect datapoints in the longitudinal direction at 1 mm far from each other. This series of data is divided into two segments of 100-mm length, and the slope of each segment is suppressed, providing a zero-mean profile. The MPD is determined as described in the ISO 13473-1 [36], while the IRI was calculated from the measured longitudinal road profile by accumulating the output of a quarter-car model and dividing by the profile length in compliance with the ASTM E1926—08 [37].

Friction coefficient measurements were performed on site with an ASFT Trailer Road CFME T5-S equipped with a smooth-tread tire. The ASFT used in this study operated at a fixed slip of 13% using a constant water film of 0.5 mm. Tests were conducted at 80 km/h, and the friction coefficient calculated every 10 m.

The International Friction Index (IFI) was developed during the PIARC experiment, and it is a standardized value used to describe the dependency of pavement surface friction on the tire sliding speed. The IFI is composed of the IFI speed number *S_p_* and friction number *F*(60), and it is based on a mathematical model called PIARC Friction Model [38]. These two numbers are computed using the following equations:

(3) $$S_{p}=a+b\cdot TX\;$$

(4) $$FR\left(60\right)=FR\left(S\right)\cdot e^{\frac{S-60}{S_{p}}}\;$$

(5) F(60)=A+B·FR(60)+C·TX 

where: *S_p_* is the IFI speed number, *TX* the macrotexture in mm, *FR(S)* the friction value at selected slip speed *S*, *FR*(60) the adjusted value of friction measurement *FR*(*S*) at a slip speed of *S* to a slip speed of 60 km/h, *a* and *b* are the calibration constants for the macro-texture measuring device (14.2 and 89.7 for this study), *A* and *B* are the calibration coefficients of the friction measuring device (0.08209 and 0.9108), and *C* the calibration number required in case a ribbed tire is used (zero for this study).

## 5. Results and Discussion

### 5.1. Rutting and Cracking Performance

**Figure 5:**
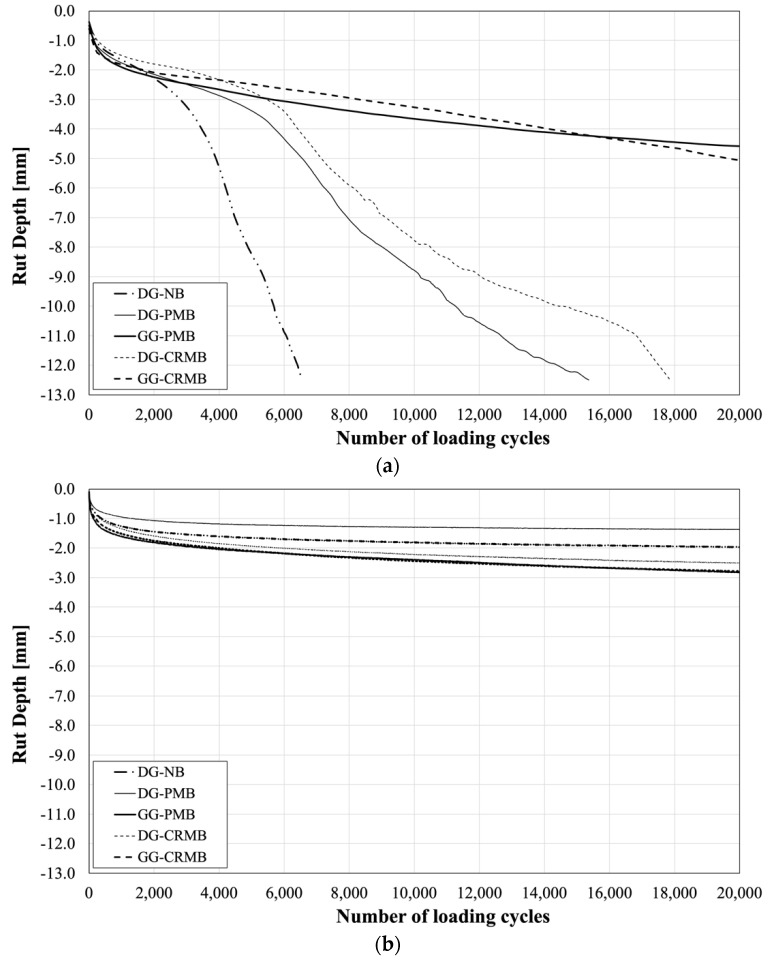
Hamburg Wheel-Tracking Test Results: (**a**) Wet and (**b**) Dry Conditions.

The results of the rutting and moisture damage characterization are presented in Figure 5a. The aggregate gradation, and the resulting job-mix formula, takes indeed the lead in response to this combination of distresses, with GG mixtures outperforming those with a DG gradation. The higher binder content of the GG HMAs, in fact, protects the aggregate particles from stripping and no moisture damage is visible at the end of the test.

Since this is the first attempt of performance-related characterization of asphalt mixtures in the UAE, no acceptance thresholds are available locally. The Texas DoT recommends an accumulated rut depth of less than 12.5 mm after 20,000 loading cycles for PG 76 or higher. If this threshold is applied in this study, only GG mixtures could be declared acceptable. However, the behavior of the HMAs in dry conditions (Figure 5b) indicates an overall good performance of all mixtures, with a slightly higher total permanent deformation accumulated after 20,000 loading cycles of the GG mixtures.

A better indication of the asphalt mixtures response in the rutting region, for both dry and wet conditions, is provided in Table 3. The Creep Slope (CS) calculated for materials tested in the presence of water indicate that, among mixtures with the same gradation, the potential differences introduced by the polymer modification seen in Figure 2 are eliminated. On the other side, when tests are performed in dry conditions, the ranking among the materials is in accordance with the results of the MSCR tests.

**Figure 6:**
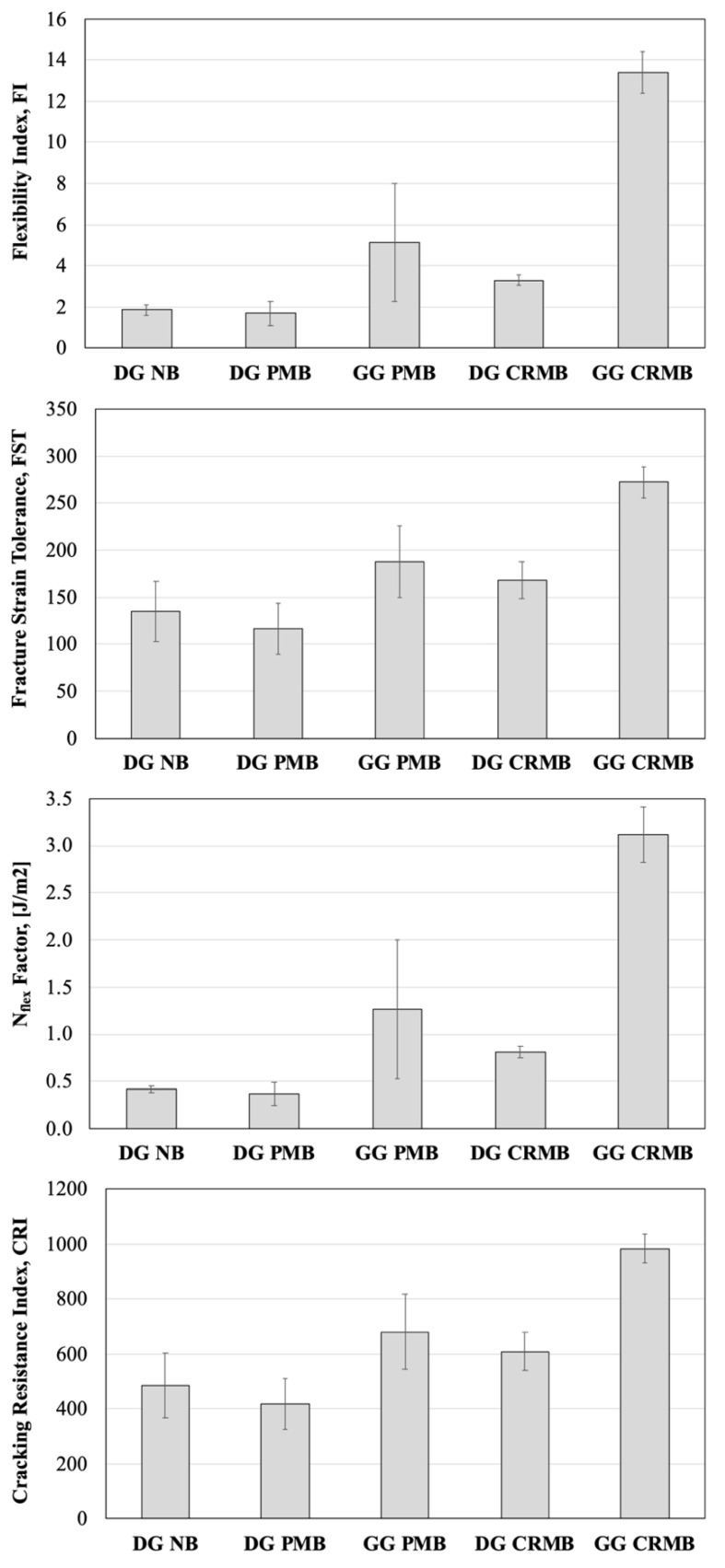
SCB Fracture Energy-Related Parameters.

The results of the SCB tests are provided in Figure 6. The GG mixes performed better in terms of cracking resistance with respect to the DG HMAs. Such a difference can be attributed to the higher percentage of bitumen rather than the gradation itself. It is worth noticing that, in agreement with the results of the bitumen characterization and regardless of the specific fracture energy-related parameter considered for comparison, it is evident that the enhanced performance of the rubberized bitumen improves the response of the HMAs.

**Figure 7:**
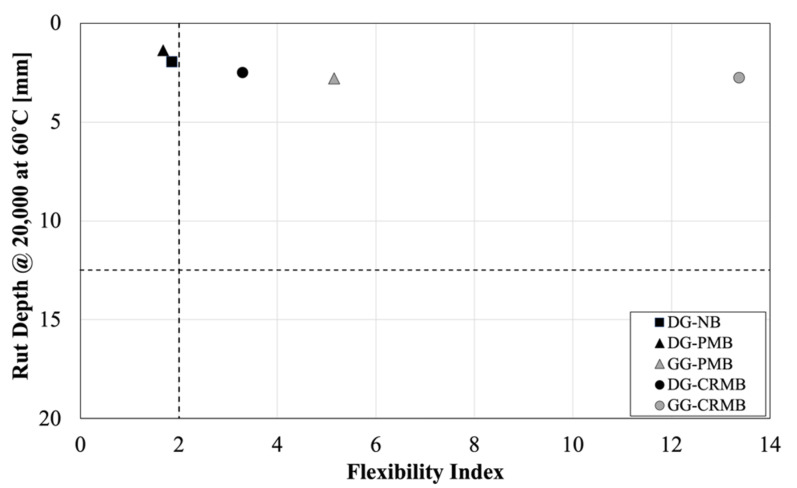
Performance-Space Diagram.

The performance-space diagram representation has been recently proposed in the framework of the balanced mix-design approach for the combined evaluation of either high-/low-temperature or high-/intermediate-temperature performance of asphalt mixtures. For this study, a comparison of the high-/intermediate-temperature performance of each mixture is reported in Figure 7 by using the HWT rut depth at 20,000 loading cycles and the SCB Flexibility Index.

It must be noticed that the thresholds (rutting depth of 12.5 mm and Flexibility Index equal to 2) have been selected from the available literature and shall be adjusted to the local conditions in future research studies. Nonetheless, the location of the data points on the diagram, regardless of the thresholds, indicates that the use of a lower binder content, especially in DG mixes, overachieves the goal of reducing permanent deformation while cracking becomes a major concern. Even though the use of rubberized bitumen can mitigate this issue in DG HMAs, cracking at intermediate temperature still remain a major concern. SCB tests are performed on short-term aged HMA, but the data points in Figure 7 would shift on the left if tests are performed on long-term aged materials, which is the typical condition for such a distress. Hence, the DG-CRMB would be positioned either borderline or below the threshold in the performance-space diagram. The implementation of a performance-based mix design could be beneficial in this scenario.

### 5.2. Sound Absorption, Roughness and Friction

**Figure 8:**
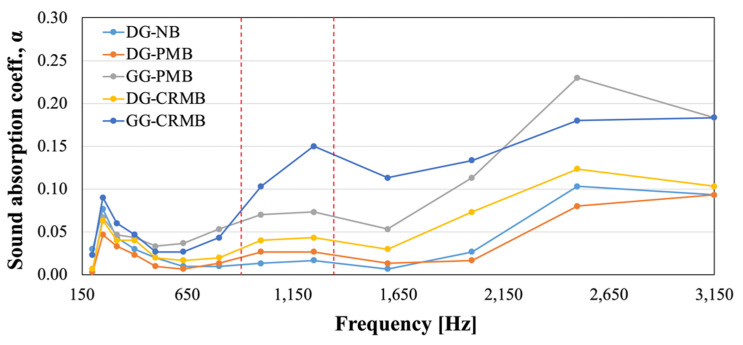
Sound Absorption Coefficients of Test Section Mixtures.

The sound absorption coefficients are reported in Figure 8 for the five mixtures tested. As indicated by Buratti and Moretti [39], the traffic noise spectra of different vehicles at different speeds have peaks between 900 and 1300 Hz. In this range, the best performing material is, undoubtedly, the GG-CRMB mixture with a sound absorption coefficient of 0.15. Although the magnitude of the coefficients might be considered low compared to other materials presented in literature, results show some benefits of using rubberized bitumen and, in general, the gap-graded gradation. Currently, DG mixes are used in the area and their sound absorption coefficients are lower than 0.1. On the other side, the GG gradation, together with a binder content lower than what commonly used for GG HMAs, create interconnected air voids that improve the noise reduction performance by up to 275% (DG-CRMB vs. GG-CRMB).

**Figure 9:**
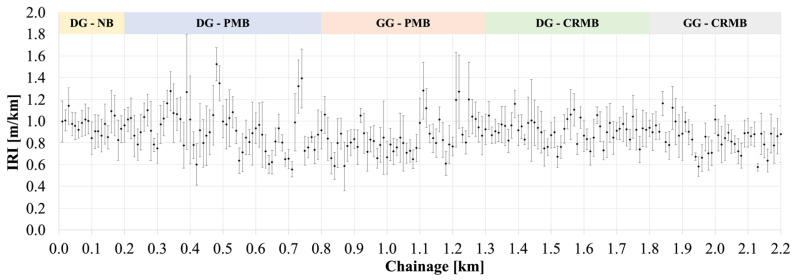
IRI Values for the Various Field Test Sections.

Figure 9 reports the IRI values calculated every 10 m over the entire length of the test section. Following the classification given by Sayers et al. in 1986 [40], IRI less than 2 m/km are acceptable for “super-highways” while values in the range 1.5–3.5 m/km are acceptable for all other new pavements. The data calculated for all test sections allow us to classify all pavement sections in the first category. It shall be reminded that the analysis was performed on the test sections after 6 milion ESALs, which still represents an early stage of pavement service life

**Figure 10:**
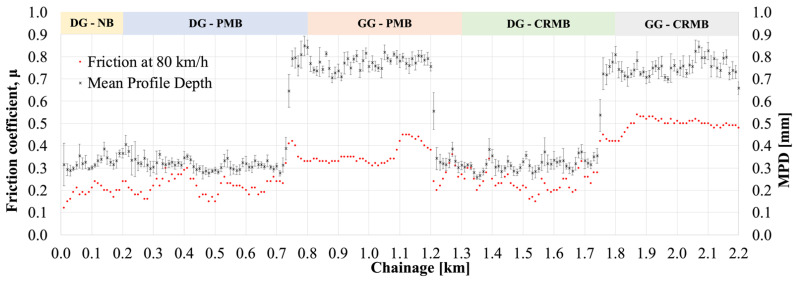
Mean Profile Depth and Friction Coefficients for the Various Sections.

Figure 10 shows the relationship between the MPD and friction coefficient for the different sections. The shifting chainage between the dense- and gap-gradation is evident from the MPD datapoints. As expected, all DG mixtures have comparable mean profile depth (0.3 mm) and, consequently, similar friction coefficients (average of 0.19, 0.22, and 0.24 for DG-NB, DG-PMB, and DG-CRMB, respectively). It is worth noticing however that the two GG mixes have comparable MPD (0.75 mm), but different friction coefficients (0.35 and 0.46 for GG-PMB and GG-CRMB, respectively), even though the only difference between the mixtures is the presence of the rubber in the modified binder.

**Figure 11:**
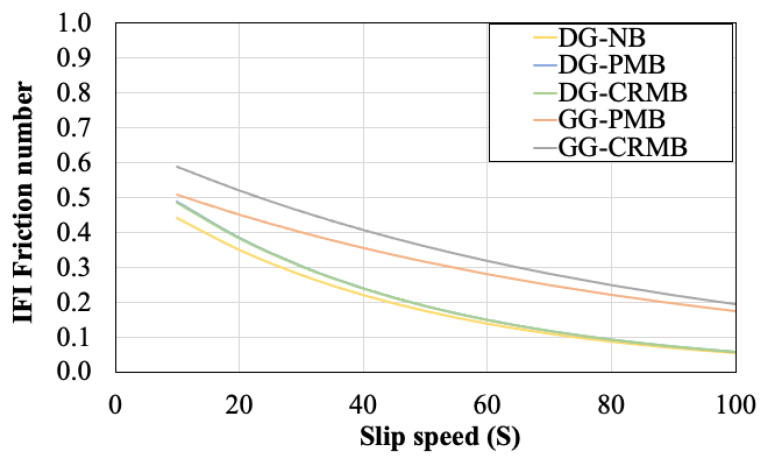
IFI friction models.

The application of the PIARC Friction Model (Equations (3)–(5)) resulted in the IFI numbers in Table 4. The benefit of using a gap-graded gradation of the aggregate is well summarized by the value of *S_p_* which represents a measure of the macro-texture influence on surface friction. Moreover, the normalized friction values of both GG HMAs were found to be double of those calculated for the DG mixes. Using the exponential function reported in Equation (4), the IFI friction number was calculated at different slip speeds as illustrated in Figure 11. Even though the difference between curves of the two GG mixes tends to decrease by increasing the slip speed, the mixture with rubberized asphalt still provides the best performance among all HMAs considered in this study. As per the results of the IRI, also for the friction values it is worth to remind that the test section was analyzed in an early stage of service life. Continuous monitoring will be performed in the years to come.

## 6. Conclusions

This paper presented the results of a research project aimed at investigating the potential benefits and drawbacks of using waste rubber from end-of-life tires in arid climate without altering the existing local requirements for mix-design. A full-scale test section was designed and constructed on a major truck-road where dense- and gap-graded mixtures produced with neat, SBS-, and CR-modified binders were sampled. The main findings of the study can be summarized as follows:

- GG mixtures were designed targeting the air voids, VMA, and VFA specific ranges of the Abu Dhabi standards. The resulting optimum binder content was out of specifications and at least 1% higher than the values obtained for DG HMAs. No issues were encountered in the design of the DG CRMB mixes.
- GG mixtures outperformed those with a dense-graded gradation when tested in the Hamburg Wheel Tracking device. The higher binder content of the GG HMAs protected aggregate particles from stripping and no moisture damage was detected. Stripping issues were visible for DG mixes already at an early stage of the test.
- Among mixtures with the same gradation, the low-binder quantity eliminates the potential performance variations due to different polymer modifications.
- For both DG and GG mixtures, the enhanced cracking resistance of the CRMB documented through rheological characterization did show up in the SCB test results too. As noticed for the moisture damage characterization, GG mixes performed better than DG due to the higher bitumen content.
- The local volumetric requirements for mix-design, and the typical resulting lower binder content, overachieves the goal of reducing permanent deformation while cracking becomes a major concern. The implementation of a performance-based mix design could be beneficial in this scenario.
- Sound absorption coefficients indicated beneficial effects due to rubberized bitumen in combination with a gap-graded gradation. Compared to mixtures currently used in the area, the combination of aggregate gap gradation and rubberized bitumen can reduce traffic noise in the field.
- As expected, significant differences were noticed between DG and GG mixtures in terms of both mean profile depth and friction coefficient. Among mixtures with the same gradation, differences were reported for GG mixes. Despite having comparable MPD, the friction coefficient of the GG-CRMB section was 30% higher than the one recorded in the GG-PMB section even though the only difference between the mixtures is the presence of the rubber in the modified binder. These results shall be intended as distinctive of an early stage of the pavement service life. The test section will be subjected to continuous monitoring in the years to come.

## Figures and Tables

**Table 1:** Physical characteristics of aggregates.

	Class of Aggregates		
	0/5 mm	5/10 mm	10/15 mm
Sand equivalent [%]	57	-	-
Soundness [%]	3	2	-
Fractured surface [%]	-	100	100
Flakiness index [%]	-	19	12
Los Angeles index [%]	-	16	9
Elongation index [%]	-	-	18
Absorption [%]	-	0.5	0.5

**Table 2:** Volumetric and mechanical Characteristics at the Mix-design Level.

	Specs	Dense-Graded			Gap-Graded	
	Limits	NB	PMB	CRMB	PMB	CRMB
Design Binder Content [%]	3.0–4.0	3.8	3.9	4.1	5.2	5.5
Air Voids [%]	5–7	6.4	6.2	6.4	6.4	6.5
VMA [%]	min. 13	15.1	15.1	15.6	18.1	19
VFA [%]	50–65	57	59	60	64	66
Marshall Stability [kN]	min. 1600	1910	2100	1680	1450	1190
Flow [mm]	2–4	2.74	3.06	3.30	4.7	3.8
Marshall Stiffness [kN/mm]	min. 550	697	686	509	309	313

**Table 3:** Creep Slope (CS) in Wet and Dry Conditions.

	Dense-Graded			Gap-Graded	
	NB	PMB	CRMB	PMB	CRMB
Creep-slope—Wet conditions (× 10^−4^)	6.1 ± 0.01	3.6 ± 0.10	2.2 ± 0.05	1.1 ± 0.05	1.2 ± 0.06
Creep-slope—Dry conditions (× 10^−5^)	3.9 ± 0.60	2.0 ± 0.70	2.7 ± 0.50	3.4 ± 0.70	4.0 ± 0.60

**Table 4:** IFI numbers.

	*Sp*	*F*(60)
DG-NB	43.43 ± 2.20	0.14 ± 0.01
DG-PMB	42.23 ± 1.80	0.15 ± 0.01
DG-CRMB	42.55 ± 2.67	0.15 ± 0.01
GG-PMB	83.68 ± 2.95	0.28 ± 0.03
GG-CRMB	81.90 ± 3.20	0.32 ± 0.02

## Data Availability

The data presented in this study are available on request from the corresponding author.
